# An Improved Adaptive Median Filtering Algorithm for Radar Image Co-Channel Interference Suppression

**DOI:** 10.3390/s22197573

**Published:** 2022-10-06

**Authors:** Nuozhou Li, Tong Liu, Hangqi Li

**Affiliations:** College of Navigation, Dalian Maritime University, Dalian 116026, China

**Keywords:** co-channel interference, adaptive median filtering algorithm, tangential interference ratio, between-class variance

## Abstract

In order to increase the accuracy of ocean monitoring, this paper proposes an improved adaptive median filtering algorithm based on the tangential interference ratio to better suppress marine radar co-channel interference. To solve the problem that co-channel interference reduces the accuracy of radar images’ parameter extraction, this paper constructs a tangential interference ratio model based on the improved Laplace operator, which is used to describe the ratio of co-channel interference along the antenna rotation direction in the original radar image. Based on the idea of between-class variance, the tangential interference ratio threshold is selected to divide co-channel interference into high-ratio regions and low ones. Moreover, an improved adaptive median filter is used to process regions of high ratio based on the median of sub-windows, while that of low-ratio regions is processed by the adaptive median filter based on the median of current windows. Radar-measured data from Bohai Bay, China are used for algorithm validation and experimental results show that the proposed filtering algorithm performs better than the adaptive median filtering algorithm.

## 1. Introduction

Relying on the advantages of detection in low-visibility conditions, marine radar is regarded as one of the key instruments in the future of ocean monitoring, pilotage and collision avoidance [1]. Especially in terms of ocean information extraction, if reliable winds [2], waves [3], currents [4], tides [5], sea ice [6] or oil spill [7] measurements can be obtained from marine radars, the costs associated with traditional field means such as anemometers, buoys or manual visual inspection can be significantly reduced [8]. Therefore, marine radar is considered to be one of the key means of ocean information extraction today, and the land-based or shipborne marine radar have received extensive attention. However, the co-channel interference signal is of high strength, which reduces the quality of radar images and affects the accuracy of echo information extraction from the radar images. Since it is spread throughout the entire radar image, accurate suppression of the co-channel interference has become a challenge under the trend of an increasing demand for ocean monitoring.

In the processing of co-channel interference, domestic and foreign scholars have proposed many methods. Generally speaking, the methods of co-channel interference removal can be divided into hardware and software methods. The hardware approach suppresses the interference signal by adjusting the transceiver design parameters [9,10,11,12,13]. The authors of [9] proposed a coherent front-end based on the phase-synchronous optoelectronic oscillator (OEO) with low phase noise; the SNR of the echo pulses has an improvement relative to the signal before pulse accumulation. In [10], a new radio frequency interference (RFI) mitigation method based on joint fractional Fourier transform (FRFT) and complex empirical mode decomposition (CEMD) is proposed. A standard component within the radio frequency (RF) radar receiver is proposed in [11] to suppress the co-channel interference. In [12], an improved histogram method for pulse repetition interval (PRI) deinterleaving based on pulse correlation is proposed, with the aim of radar pulse deinterleaving. However, the frequency band selection of radars is subject to many factors. The effect of the methods above is greatly reduced when all radar frequency bands are occupied. In addition, when two ships are close to each other, the effect on co-channel interference suppression is limited [13].

The software methods mainly include mean filtering, median filtering, adaptive filtering, Wiener filtering and filtering based on wavelet transform [14,15,16,17,18,19,20]. In [14], an improved adaptive mean filtering algorithm is proposed by assigning a certain weight to the pixel gray values of the noise points in the filter template, which can effectively remove pulse noise points. The authors of [15] proposed a non-local mean (NLM) filtering algorithm to enhance image quality in a highly turbid environment. In [16], a fully 3D non-local mean parallel approach is designed and implemented. To demonstrate its high applicability and scalability, different algorithm mapping strategies on a GPU architecture and multi-GPU framework are adopted. In [17], the computational model of weighted mean filtering and the characteristics of a high-performance computer architecture are studied. Moreover, an efficient hierarchical image-weighted mean filtering parallel algorithm for Open Computing Language (OpenCL) is designed and implemented. According to the respective correlation matrices of images and noise, many works have been conducted on the Wiener filter [18,19,20]. The purpose of [18] is to confirm that image quality can be improved by the median modified Wiener filter (MMWF) technique, and the authors of [19] proposed a synthetic aperture radar (SAR) image anti-speckle filter based on an extended adaptive Wiener filter (EAWF), extended guided filter (EGF) and weighted least squares (WLS) filter. In [20], contrast-limited adaptive histogram equalization (CLAHE) is used to improve contrast, and the Wiener filter is used for noise reduction. Adaptive filtering uses the filter parameter results obtained at the previous moment to automatically adjust those at the present moment to achieve optimal filtering [21,22,23,24]. In [21], the formalism for combining the Bayesian maximum entropy strategy with the variational analysis (VA) paradigm is presented to improve the Bayesian maximum entropy–variational analysis (BMEVA) performance for high-resolution radar imaging and denoising. The feasibility of integrating an adaptive filter approach for the compensation of platform motion artefacts is investigated in [22] for the extraction of respiratory motion signatures. In [23], an algorithm based on the fuzzy impulse detection technique is proposed, which can remove impulse noise efficiently from highly corrupted images while preserving image details. The authors of [24] proposed a novel adaptive Type-2 fuzzy filter for removing salt and pepper noise from images. In this study, two approaches have been proposed for finding the threshold between different types of pixels by designing a primary membership function (MF). The above software-based methods eliminate image interference or noise to a certain extent, but they still have room for improvement in image target protection or computational speed.

In this paper, we consider an improved adaptive median filtering algorithm based on the tangential interference ratio for radar co-channel interference suppression. In this work, we aim to automatically identify regions of different interference ratios to optimize the selection of filtering windows. The major contributions of this paper are as follows:

- Based on the source of radar co-channel interference and the storage form of radar echo data, the Laplace operator is improved to better identify radial interference.
- Radar images are processed with the improved Laplace operator and binarized to establish the tangential interference ratio model. Based on the idea of between-class variance, the tangential interference ratio threshold is determined, which provides a classification basis for different filtering methods in high-ratio regions and low ones.
- We explore the improved adaptive median filtering algorithm, using different filtering windows for high-ratio regions and low ones. On the basis of protecting the details of radar echo images to the maximum extent, the gray values of interference points are replaced with the median of pixel points in the adaptive window.

The remainder of this paper is organized as follows. Section 2 introduces the improved method of Laplace operator based on the radar co-channel interference source and radar echo data storage form. Section 3 explains the principles of the OTSU algorithm firstly, and then introduces the construction process of the tangential interference ratio model and finally gives the algorithm flow. The effectiveness of the proposed algorithm is provided in Section 4, and the conclusion is given in Section 5.

## 2. Improvement of Laplace Operator

### 2.1. Characteristics of Co-Channel Interference

During the working process of a marine radar, it will be interfered with by the electromagnetic waves emitted by other nearby radars in the same frequency band, resulting in the generation of radial interference on the radar grayscale images—that is, co-channel interference. Co-channel interference can be divided into co-channel synchronous interference and co-channel asynchronous interference.

(1) $$\Delta t=\left|\frac{1}{PRF_{1}}-\frac{1}{PRF_{2}}\right|$$

where $PRF_{1}$ and $PRF_{2}$ denote the pulse repetition frequency transmitted by the two radars, respectively. $T_{1}$ and $T_{2}$ represent the pulse widths of the two radars, respectively; let Δt be given by (1).

When $\Delta t\leq T_{1}$, the interference of the first radar with the other one is known as co-channel synchronous interference, and vice versa as co-channel asynchronous interference [25]. This paper mainly discusses the suppression of the latter. Considering that the radar co-channel interference signal appears as a radial bright spark with obvious directionality on the echo images, this paper improves the Laplace operator, which is suitable for point detection, to become a line detection template.

### 2.2. Improvement of Laplace Operator

The Laplace operator of a two-dimensional function f(x,y) is the second-order derivative defined as follows [26]:

(2) $$\begin{array}{ccc} \nabla^{2}f\left(x,y\right) & = & \frac{\partial^{2}f\left(x,y\right)}{\partial x^{2}}+\frac{\partial^{2}f\left(x,y\right)}{\partial y^{2}}\\ & = & f(x+1,y)+f(x-1,y)-2f(x,y) \end{array}$$

where ∇ represents the direction in which the value of a continuously differentiable multivariate function increases the fastest at a certain point. ∂ is the partial derivative of f(x,y) with respect to a variable. The difference method is used to calculate second-order derivatives of the Laplace operator in the *x* and *y* directions, and the second-order difference in the two-dimensional function f(x,y) in the *x* and *y* directions can be obtained as follows [27]:

(3) $$\frac{\partial^{2}f\left(x,y\right)}{\partial x^{2}}=f\left(x+1,y\right)+f\left(x-1,y\right)-2f\left(x,y\right)$$

(4) $$\frac{\partial^{2}f\left(x,y\right)}{\partial y^{2}}=f\left(x,y+1\right)+f\left(x,y-1\right)-2f\left(x,y\right)$$

Therefore, the difference form of the Laplace operator is

(5) $$\begin{array}{ccc} \nabla^{2}f\left(x,y\right) & = & \frac{\partial^{2}f\left(x,y\right)}{\partial x^{2}}+\frac{\partial^{2}f\left(x,y\right)}{\partial y^{2}}\\ & = & f(x+1,y)+f(x-1,y)+f(x,y+1)+f(x,y-1)-4f(x,y) \end{array}$$

It is written in the form of a matrix as follows [28]:

(6) $${\mathrm{template}}_{1}=\left[\begin{array}{ccc} 0 & 1 & 0\\ 1 & -4 & 1\\ 0 & 1 & 0 \end{array}\right]$$

The matrix has the same values in the four directions of up, down, left and right. Since the co-channel interferences in the situation involved in this paper are all radial bright sparks along the radial direction, the traditional Laplace operator is not suitable because of its directionality-free characteristic.

In order to better detect co-channel interference and enhance the radial grayscale mutation, the improved Laplace operator is defined as follows:

(7) ∇2f(x,y)=3(f(x−1,y)+f(x,y)+f(x+1,y))1−32(f(x−1,y−1)+f(x,y−1)+f(x+1,y−1))1−32(f(x−1,y+1)+f(x,y+1)+f(x+1,y+1))

The template of the improved operator can be expressed as

(8) $${\mathrm{template}}_{2}=\left[\begin{array}{ccc} -1.5 & 3 & -1.5\\ -1.5 & 3 & -1.5\\ -1.5 & 3 & -1.5 \end{array}\right]$$

## 3. Improved Algorithm

The median filtering method is a nonlinear smoothing technique, which replaces the gray value of each pixel with the median value of all pixel gray values in a certain neighborhood window of the point. The principle of the median filtering method is to use a sliding template of a certain structure to sort the pixels in the template according to the size of the pixel value [29], and generate a data sequence that monotonically increases (or decreases). It is commonly used because it can remove nearly all interference or noise, with little impact on the original image. However, in addition to the large amount of computation required by the median filtering method, the original median filtering method does not have a proper distinction between interference ratios, so that it is not effective in removing co-channel interference in high-ratio regions. In this paper, the concept of the tangential interference ratio is introduced into the median filtering algorithm. The idea of between-class variance is introduced to find the threshold between high-ratio regions and low ones in the binarized image. Finally, a tangential interference ratio model is constructed to filter out radar co-channel interference.

### 3.1. Radar Image Collection and Analysis

The data in this paper were collected from the Bayuquan radar station in Yingkou, China.

**Figure 1:**
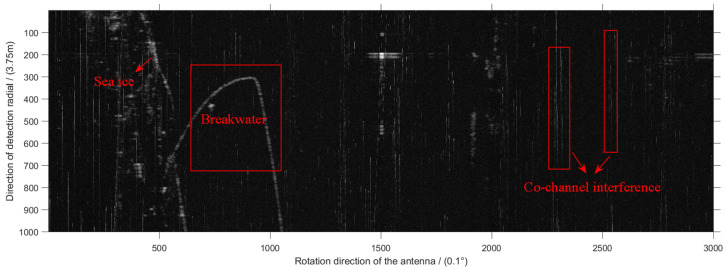
Original radar image in Cartesian coordinate system.

Figure 1 is an echo image received by the radar antenna. The irregular echoes on the left side of Figure 1 are the sea ice echoes, and the red box in the middle is the breakwater built to prevent the invasion of waves. As the research object of this paper, the radar co-channel interference signal appears as bright sparks throughout the direction perpendicular to the *X* axis, i.e., the direction of the detection radial. The two red boxes on the right of Figure 1 are examples of co-channel interference signals. The positive direction of the *X* axis represents the rotation direction of the antenna and the direction of the *Y* axis represents the direction of the detection radial. The whole image contains 1000×3000 pixels, the angular resolution of the *X* axis direction is 0.1° and the distance resolution of the *Y* axis direction is 3.75 m.

### 3.2. Median Filtering Algorithm

The echo signal received by radar antennae is often polluted by a lot of interference and noise during its formation and transmission. In addition to the Gaussian noise generated by the machine itself, there is also co-channel interference. In order to suppress and eliminate these interferences and noise, thus improving the quality of images, it is necessary to perform denoising processing on the image—that is, filtering processing.

The median filtering algorithm proposed by Tukey has been widely used to remove polluted points in images. The standard median filtering algorithm selects pixels in digital images or sequences and pixels around the adjacent pixels, and then takes the pixel value in the middle position after sorting as the pixel value of the current pixel [30]. This method allows the surrounding pixel values to be close to the true value, thereby eliminating isolated polluted points [31]. However, as the interference or noise ratio increases, the standard median filtering algorithm is not effective in preserving image details. The standard median filtering algorithm is described as follows Algorithm 1.
**Algorithm 1** Median filtering algorithm**Step 1:** Obtain first address of the original image and size of the image;**Step 2:** Open up a memory buffer to temporarily store the processing results and initialize it to 0;**Step 3:** Scan pixels in the binarized image through the loop statement, and sort the pixel values of each element in its neighborhood in ascending order. Finally, assign the obtained intermediate value to the pixel corresponding to the current point in the target image;**Step 4:** Repeat step 3 until all pixels in the original image are processed;**Step 5:** Copy running result from the memory buffer to the data region of the original image to complete filtering process.

In recent years, a variety of adaptive algorithms based on the median filtering algorithm have appeared. The extreme median filter (EM) proposed in [32] firstly divides all pixels into two categories according to the criterion: noise points and signal points. Then, the noise point is replaced by the median value of the neighborhood of that point according to the spatial correlation. The authors of [33] proposed the switching median filter (SM) algorithm, and a further optimized progressive switching filter (PSM) algorithm based on the SM algorithm is proposed in [34]. The minmax algorithm and the weighted median filter (WM) algorithm are proposed in [35,36], respectively. These algorithms have allowed useful explorations in improving the performance of median filters, but have their own limitations in practical applications because of the different interferences or noises that they deal with. The SM algorithm works well when dealing with low interference or noise conditions, but its performance gradually approaches that of the standard median filtering algorithm as the signal-to-noise ratio (SNR) of input images decreases. The PSM algorithm is a cyclic operation, which takes a long time to execute, and it has the requirement of estimating parameters in advance, which limits its real-time application. Although the minmax algorithm reduces the accumulation of propagation of error to a certain extent, the blurring of details is not well resolved. The WM algorithm reduces the loss of details by weighting, but its denoising performance also decreases.

Taking the EM algorithm as an example, the filtering method can be expressed as

(9) $$y_{ij}=\left\{\begin{array}{c} med\left(W\left[x_{ij}\right]\right),x_{ij}\in N\\ x_{ij},x_{ij}\in S \end{array}\right.$$

where $x_{ij}$ denotes each pixel of the image, *i* and *j* denote the row and column coordinates of the pixel, respectively, $W[x_{ij}]$ denotes a window of points in the image $x_{ij}$, $med(W\left[x_{ij}\right])$ means taking the median of all points in the window $W[x_{ij}]$, $y_{ij}$ is the output of image $x_{ij}$ processed by the EM algorithm, N is the set of noise points and S is that of signal points.

The EM algorithm shows advantages in both speed and performance. However, most of the improved median filtering algorithms, including the EM algorithm, are oriented to the identification of interference or noise points, rather than to the judgment of its ratio. In fact, due to the change in the ratio of co-channel interference, the existing median filtering algorithm cannot effectively remove them in high-ratio regions.

### 3.3. Model Construction

After using the improved Laplace operator to detect the co-channel interference and binarizing the original radar image $I_{1000\times 3000}$, the interference judgment matrix $B_{1000\times 3000}$ is obtained. A 60-column row vector is used as a sliding window to traverse the entire radar image, and the tangential interference ratio is stored in turn to construct a tangential interference ratio model. The interference points are divided into high-ratio regions and low ones by setting a threshold. Among this, we complete the classification with the inter-class variance. The interference ratios obtained in previous steps are divided into two categories; the within-class variance of the two categories is calculated by the method of second-order cumulative moment. The largest inter-class variance between them is then obtained by class separability measurements as the division principle of high-ratio regions and low ones. For the interference points in low-ratio regions, the adaptive median filtering algorithm is used to remove the co-channel interference. For high-ratio regions, the adaptive median filtering algorithm is further improved. Starting from the defined minimum window size, we determine whether median value of the current sub-window is an interference point. If not, we take its gray value and assign it to the current pixel point; otherwise, we expand the window size to judge again. When the last Δn pixels of the filter window are removed, this is the sub-window defined in this paper. In this process, Δn is the difference between the actual tangential interference ratio in the region and the ratio threshold. A flowchart of the improved adaptive median filtering algorithm based on between-class variance can be represented by the following figure.

After obtaining the interference judgment matrix, the first step is to construct a tangential interference ratio model. By setting a threshold, the interference points are divided into high-ratio regions and low ones. The tangential interference ratio model is a graph drawn according to the statistical law of the interference ratio in the x-direction in the Cartesian coordinate system. A 1×50 sliding window is used to traverse each row of the original radar image matrix in turn; the ratio of interference points to the pixel blocks of the window is saved in turn and then drawn into a curve graph. In this paper, we take 4.4 n miles, 5.3 n miles and 6.1 n miles to the radar detection center as examples, and the tangential interference ratio model is expressed as follows.

**Figure 2:**
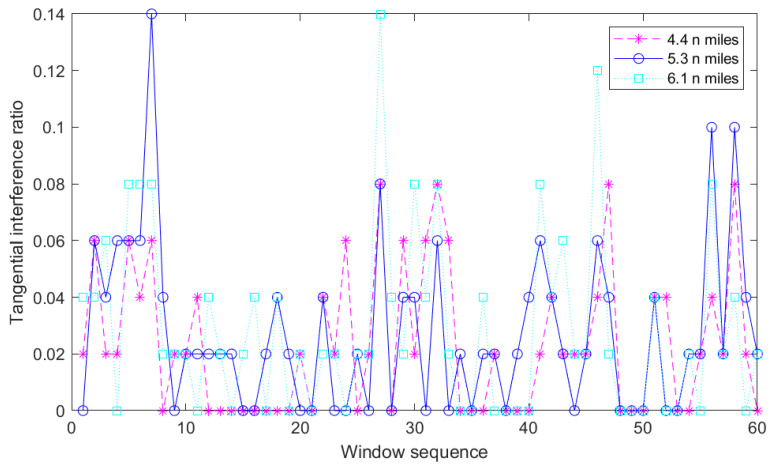
Tangential interference ratio model at 4.4 n miles, 5.3 n miles and 6.1 n miles.

The dotted line in magenta in Figure 2 represents the interference ratio everywhere along the tangential direction at a distance of 4.4 n miles. Similarly, the solid line in cyan and the dot dash line in blue represent the interference ratio at 5.3 n miles and 6.1 n miles, respectively.

**Figure 3:**
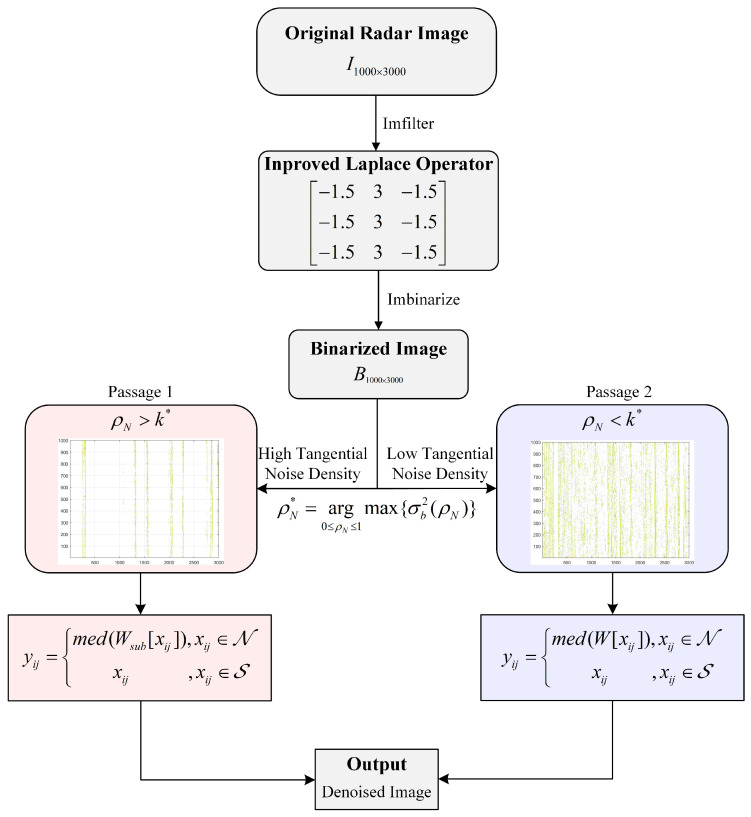
Flowchart of the improved adaptive median filtering algorithm.

In the second step, since the existing median filtering algorithm has a poor denoising effect on the dense regions of co-channel interference, this paper proposes a method based on between-class variance to find the tangential interference ratio threshold to distinguish high-ratio regions from low-ratio regions. Suppose that the tangential interference ratio obtained in the previous step is represented in ρ levels (0,1,2,⋯,ρ). Let $\rho_{i}$ denote the number of interference points at ratio level *i*, and *D* denote the total number of interference points [37]. The probability of occurrence of level *i* is given by $p_{i}=\rho_{i}/\sum_{i=0}^{\rho }\rho_{i}$. Let the tangential ratio of interference points be divided into $\rho_{L}$ and $\rho_{H}$ according to the threshold $\rho_{N}$. $\rho_{L}$ consists of interference points with a tangential interference ratio level of $\left[0,\cdots ,\rho_{N}\right]$, and $\rho_{H}$ consists of interference points with a tangential interference ratio level of $\left[\rho_{N}+1,\cdots ,\rho \right]$. The innovation of this paper is mainly aimed at the processing of the points with a level of $\rho_{H}$. Let $P_{H}\left(\rho_{N}\right)$ and $P_{H}\left(\rho_{N}\right)$ denote the cumulative probability. $\mu_{L}\left(\rho_{N}\right)$ and $\mu_{H}\left(\rho_{N}\right)$ represent the mean levels. $\sigma_{L}^{2}\left(\rho_{N}\right)$ and $\sigma_{H}^{2}\left(\rho_{N}\right)$ denote the variances of the classes $\rho_{L}$ and $\rho_{H}$, respectively. The specific calculation rule is given as Figure 3.

The probability of occurrence of a interference point with a tangential interference ratio $\left[0,\cdots ,\rho_{N}\right]$ can be given by

(10) $$P_{L}\left(\rho_{N}\right)=\sum_{i=0}^{\rho_{N}}p_{i}$$

Similarly, the probability of occurrence of a interference point with a tangential interference ratio $\left[\rho_{N}+1,\cdots ,\rho \right]$ is

(11) $$P_{H}\left(\rho_{N}\right)=\sum_{i=\rho_{N}+1}^{\rho }p_{i}=1-P_{L}\left(\rho_{N}\right)$$

We divide the tangential interference ratio into $\rho_{L}$ and $\rho_{H}$, according to the knowledge of probability theory [38]:

(12) $$\mu =P_{L}\left(\rho_{N}\right)\mu_{L}\left(\rho_{N}\right)+P_{H}\left(\rho_{N}\right)\mu_{H}\left(\rho_{N}\right)$$

where $\mu_{L}\left(\rho_{N}\right)$ and $\mu_{H}\left(\rho_{N}\right)$ are the mean values of these two types of tangential interference ratio regions, respectively, and μ is the global mean value.

According to the concept of variance, the expression of between-class variance is

(13) $$\begin{array}{ccc} \sigma_{b}^{2}\left(\rho_{N}\right) & = & P_{L}\left(\rho_{N}\right){\left(\mu_{L}\left(\rho_{N}\right)-\mu \right)}^{2}+P_{H}\left(\rho_{N}\right){\left(\mu_{H}\left(\rho_{N}\right)-\mu \right)}^{2}\\ & = & P_{L}\left(\rho_{N}\right)P_{H}\left(\rho_{N}\right){\left(\mu_{L}\left(\rho_{N}\right)-\mu_{H}\left(\rho_{N}\right)\right)}^{2} \end{array}$$

According to Equation (13), we traverse each tangential interference ratio obtained in the first step in turn [39]. When $\sigma_{b}^{2}\left(\rho_{N}\right)$ takes the maximum value $\max\{\sigma_{b}^{2}\left(\rho_{N}\right)\}$, the threshold of the high and low tangential interference ratio regions $\rho_{L}$ and $\rho_{H}$ is defined. The algorithm used to accomplish this task is as follows Algorithm 2.
**Algorithm 2** Tangential interference ratio model construction**Step 1:** Obtain first address of the original image and size of the image;**Step 2:** Open up a memory buffer to temporarily store the processing results and initialize it to 0;**Step 3:** Obtain the binarized image and scan it with a certain row vector window; save the ratio of interference points in the window;**Step 4:** Scan pixels in the binarized image through the loop statement;**Step 5:** Repeat step 4 until all pixels in the binarized original image are processed; using the maximum between-class variance method to find the threshold of the interference ratio, the obtained interference points are divided into high-ratio regions and low ones.

By calculation, $\max\{\sigma_{b}^{2}\left(\rho_{N}\right)\}$ = 9.8% is the tangential interference ratio threshold selected in this paper. The high and low tangential interference ratio regions segmented by this threshold are as follows.

**Figure 4:**
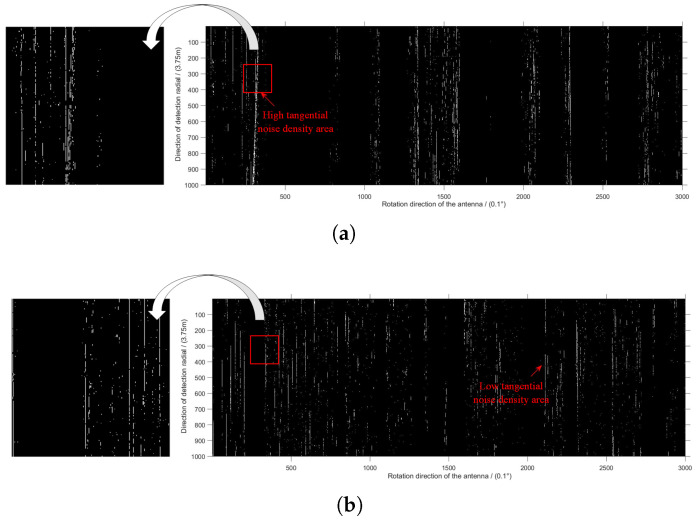
Comparison of high and low tangential interference ratio regions. (**a**) High tangential interference ratio regions, (**b**) low tangential interference ratio regions.

The bright sparks in Figure 4a,b represent regions of high and low tangential interference ratio, respectively. In order to better understand Figure 4a,b, a part of the original image has been extracted and displayed on the left side of the corresponding picture. The left side of Figure 4a is the tangential interference points with interference ratio greater than 9.8%, while Figure 4b is the opposite.

### 3.4. Improvement of Median Filtering Algorithm

The method described in this section is optimized for regions of high tangential interference ratio in Section 3.3.

Due to the characteristics of high tangential interference ratio regions being greatly interfered with by the other interference in the window during the median filtering process, the adaptive median filtering in the window cannot fundamentally improve the interference removal effect. This section reduces the interference by adaptively changing the elements in the window. The core of the traditional median filtering algorithm is $y_{ij}=med\left(W\left[x_{ij}\right]\right)$ when a point is judged to be an interference or noise point [40]. This paper sorts the gray value elements in the selected window firstly to obtain $Sort(W\left[x_{ij}\right])$. Then, the last Δn elements in the window are removed, where Δn is the difference between the tangential interference ratio at this point and the ratio threshold $\rho_{N}$. The new window obtained after this step is defined as $W_{sub}\left[x_{ij}\right]$ in this paper. This step draws most of the high-brightness interference in the window to the outside, thus greatly avoiding its influence on the filtering effect.

**Figure 5:**
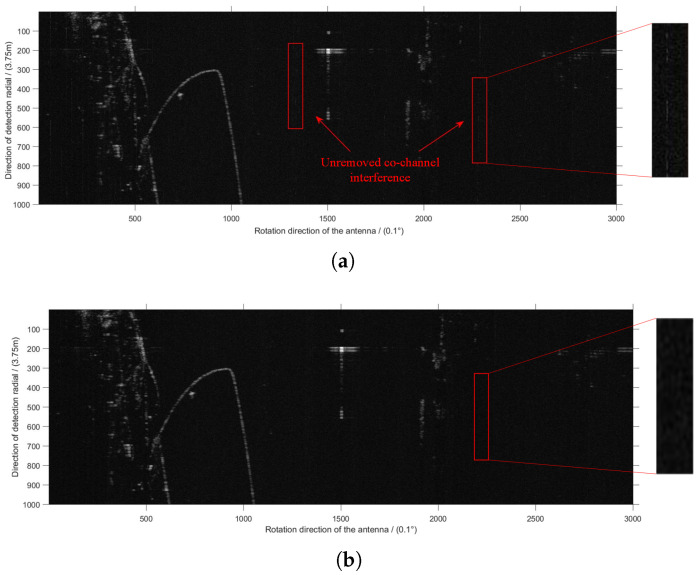
Comparison of processing results. (**a**) Results of adaptive median filtering algorithm, (**b**) results of the proposed algorithm.

Figure 5a is the processing result of the adaptive median filtering algorithm. The method in this paper can effectively suppress more co-channel interference, such as the part highlighted by the arrow in Figure 5a, thus showing that the improved adaptive median filtering algorithm has better performance.

## 4. Experiment and Simulation

In this paper, several commonly used image quality evaluation parameters are used to evaluate the advantages and disadvantages of the improved algorithm and several other filtering methods.

Let f(x,y) be the original radar image signal received by the radar receiver, and $\hat{f}\left(x,y\right)$ be the image signal processed by different algorithms. The Root Mean Square Error (*RMSE*) represents the average error between image pixels [41], which is defined as

(14) $$RMSE=\sqrt{\frac{1}{H\times W}\sum_{x=1}^{H}\sum_{y=1}^{W}{\left(f\left(x,y\right)-\hat{f}\left(x,y\right)\right)}^{2}}$$

where *H* and *W* represent the size of the image data.

**Figure 6:**
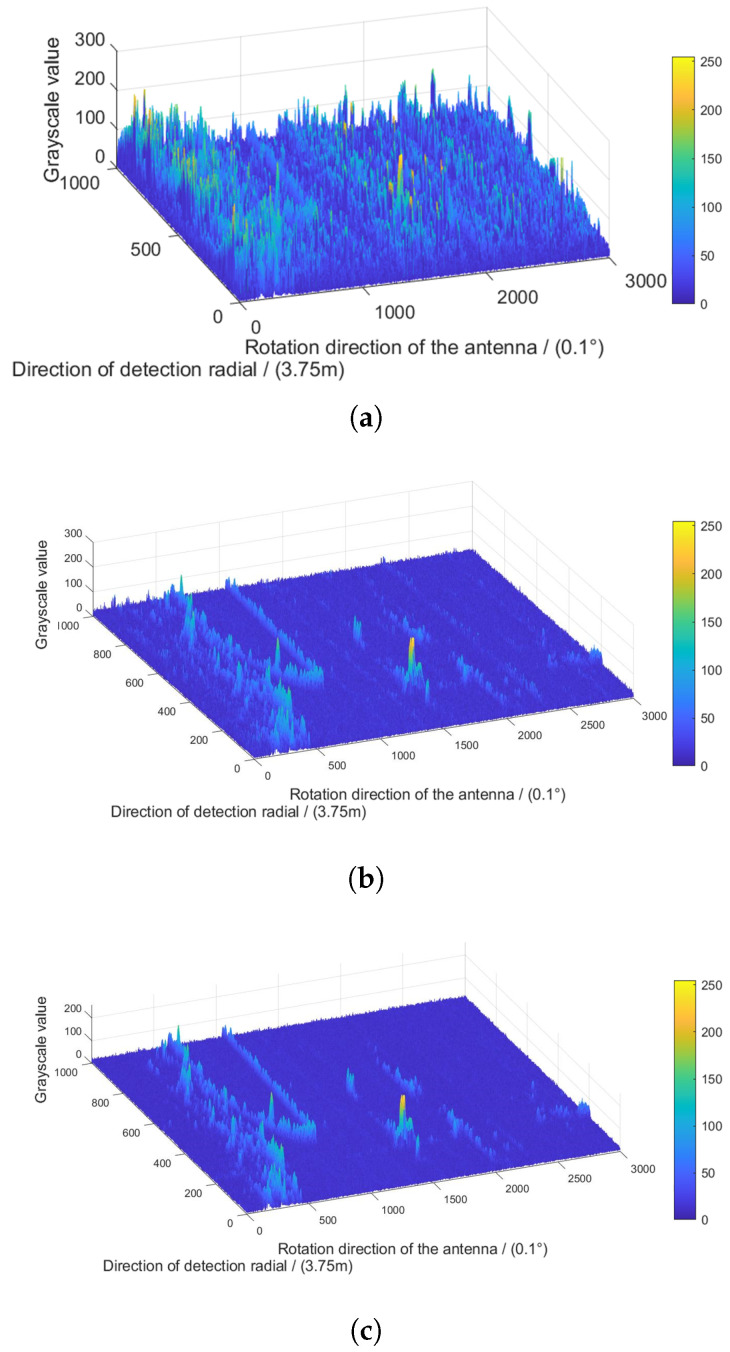
Comparison of mesh surface graphs between algorithms. (**a**) Mesh surface graph of original radar image, (**b**) mesh surface graph of the image processed by the adaptive median filtering algorithm, (**c**) mesh surface graph of the image processed by the proposed algorithm.

The mesh surface graphs of the original radar image, the image processed by the adaptive median filtering algorithm and that processed by the algorithm in this paper are represented in Figure 6a–c.

Compared with the adaptive median filtering method, the *RMSE* of the method in this paper is reduced from 3.8060 to 2.0561 by (14). The proposed algorithm removes some interference points in the filter window and optimizes the selection of the median, whereas the *RMSE* is sensitive to outliers in data. Therefore, through calculation, the proposed algorithm reduces the *RMSE* of the radar image by 1.7499, which improves the image quality.

Peak Signal to Noise Ratio (*PSNR*) is the most common and widely used image objective evaluation index; it is based on the error between corresponding pixels, i.e., an error-sensitive image quality evaluation [42]. The *PSNR* is expressed as follows:

(15) $$PSNR=10\times \log_{10}\frac{65025}{RMSE}$$

where PSNR is a measure of image quality. An increase in *PSNR* represents higher image quality. Compared with the adaptive median filtering method, the *PSNR* of the algorithm in this paper increased from 42.3 to 45 by (15). It can be seen that the proposed algorithm can better remove the co-channel interference in the radar image and improve the image quality.

When the Signal to Noise Ratio (*SNR*) is used to judge the quality of filtered images, the larger its value is, the more thorough the image filtering is. *SNR* is also a key indicator for evaluating image filtering quality, which can be given by

(16) $$SNR=10\times \log_{10}\frac{\sum_{x=1}^{H}\sum_{y=1}^{W}\hat{f}{\left(x,y\right)}^{2}}{\sum_{x=1}^{H}\sum_{y=1}^{W}{\left(f\left(x,y\right)-\hat{f}\left(x,y\right)\right)}^{2}}$$

By calculation, the radar images processed by the adaptive median filtering algorithm and the proposed algorithm have *PSNR*s of 10.8453 and 15.9134, respectively. The higher *SNR* indicates that the improvement of the proposed algorithm is more suitable for radar co-channel interference.

In order to prove the universality and effectiveness of the proposed algorithm in suppressing radar co-channel interference, the adaptive median filtering algorithm and the proposed algorithm are used to filter 20 radar images, and the *RMSE*, *PSNR* and *SNR* are calculated, as shown in Table 1. In order to better verify the effectiveness of method in this paper, we draw SNR line graphs of images processed by the adaptive median filtering algorithm and the proposed algorithm under different tangential interference ratios.

**Figure 7:**
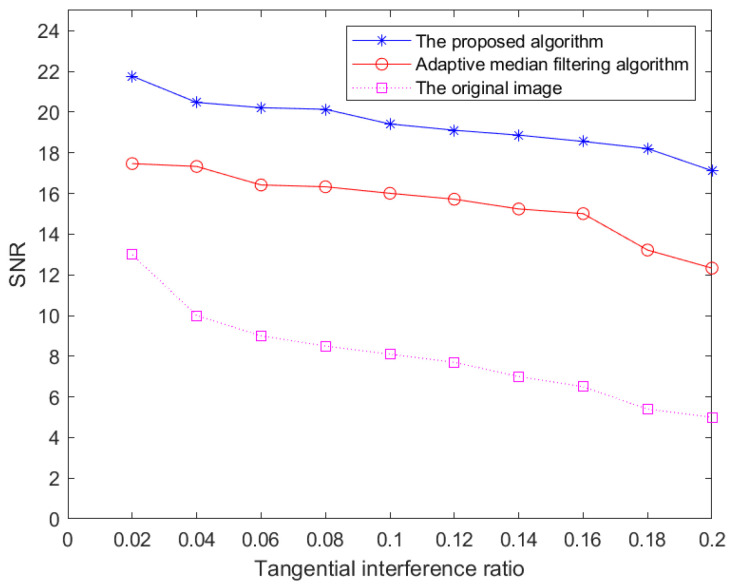
Relationship between *SNR* and tangential interference ratio of images processed by different algorithms.

It can be seen from Table 1 and Figure 7 that the *PSNR* and *SNR* of the algorithm in this paper are both larger than those of the adaptive median filtering algorithm, which shows that the former is more thorough in filtering and has less impact on the ocean information of the original image. In addition, the effect of the proposed algorithm is more obvious as the tangential interference ratio increases.

Therefore, it can be concluded that the improvement of the adaptive median filtering algorithm in this paper is better than the algorithm before improvement, as it can deal with co-channel interference well and maintain the original ocean information. This improved image feature can be adapted to the later extraction of ocean information, especially the extraction of weak echo signals such as sea ice echoes.

## 5. Conclusions

The application of the median filtering algorithm and its various improved algorithms in the software removal of radar co-channel interference is studied. By improving the Laplace operator, a line detection template is formed, thereby enhancing the radial grayscale mutation of the radar image. In the filtering of interference points, the algorithm in this paper inherits the advantages of the adaptive median filtering algorithm. At the same time, the proposed algorithm establishes a tangential interference ratio model and determines the ratio threshold based on maximum between-class variance. For interference points in low-ratio regions, we replace the values of interference points with the median of current windows. Moreover, the median of the current window after removing the last Δn elements is used to replace the value of interference points in high-ratio regions, so as to prevent the gray value of the dense interference point from interfering with the selection of the median value.

Results show that the proposed algorithm can effectively remove the co-channel interference in original radar image data. The *RMSE* of the image processed by the improved algorithm dropped from 3.8060 to 2.0561, while both *PSNR* and *SNR* increased. It is proven that the proposed algorithm has a more obvious effect on removing the co-channel interference, and has less influence on the useful signal of original images. The proposed algorithm can also be applied to the removal of interference or noise in other situations. For further research, due to the complexity of sea conditions, research based on the proposed algorithm and the echo characteristics of rain, snow and waves would be meaningful. 

## Figures and Tables

**Table 1:** Comparison of *RMSE*, *PSNR* and *SNR* between algorithms.

Number	Adaptive Median Filtering Algorithm			Improved Algorithm		
	*RMSE*	*PSNR*	*SNR*	*RMSE*	*PSNR*	*SNR*
1	3.2669	42.9895	11.7073	2.2625	44.5849	14.3718
2	3.3671	42.8582	11.4970	2.2543	44.6008	17.4794
3	3.4815	42.7131	11.4867	2.1845	44.7372	15.1643
4	3.5736	42.5998	11.2664	2.1168	44.8740	15.5058
5	3.7216	42.4235	11.2098	2.1660	44.7742	15.6986
6	3.7948	42.3389	10.8543	2.1527	44.8009	15.4729
7	3.8089	42.3228	10.9757	2.2140	44.6791	15.4185
8	3.8290	42.3000	10.6038	2.1050	44.8984	15.4398
9	3.8306	42.2981	10.6602	1.9375	45.2584	16.2738
10	3.9080	42.2112	10.6859	2.1595	44.7872	15.5649
11	3.9200	42.1979	10.8312	1.8700	45.4124	17.0951
12	3.9502	42.1646	10.8808	1.7030	45.8187	18.0732
13	3.9571	42.1570	10.6083	1.8101	45.5537	17.2207
14	3.9592	42.1548	10.3614	1.8407	45.4809	16.7618
15	3.9642	42.1493	10.2517	2.3126	44.4897	14.5196
16	3.9912	42.1198	10.3289	1.8404	45.4818	16.8225
17	3.9947	42.1160	10.7881	1.8982	45.3747	17.1216
18	4.0455	42.0611	10.4583	1.7304	45.7493	17.6863
19	4.0469	42.0596	10.2852	1.7037	45.8168	17.6102
20	4.0696	42.0353	10.4553	1.7924	45.5965	17.4258

## Data Availability

The data presented in this study are available on request from the corresponding author.
